# Histone chaperone HIRA deposits histone H3.3 onto foreign viral DNA and contributes to anti-viral intrinsic immunity

**DOI:** 10.1093/nar/gkx771

**Published:** 2017-09-13

**Authors:** Taranjit Singh Rai, Mandy Glass, John J. Cole, Mohammad I. Rather, Morgan Marsden, Matthew Neilson, Claire Brock, Ian R. Humphreys, Roger D. Everett, Peter D. Adams

**Affiliations:** 1 Institute of Biomedical and Environmental Health Research, University of the West of Scotland, Paisley, PA1 2BE, Scotland; 2 Beatson Institute for Cancer Research, Glasgow, Scotland; 3 Institute of Cancer Sciences, University of Glasgow, Glasgow, G61 1QH, Scotland; 4 MRC-University of Glasgow Centre for Virus Research, University of Glasgow, Glasgow, G61 1QH, Scotland; 5 Cardiff Institute of Infection & Immunity, Cardiff University, Cardiff, Wales, CF14 4XN, UK; 6 Sanford Burnham Prebys Medical Discovery Institute, La Jolla, CA 92037, USA

## Abstract

The HIRA histone chaperone complex deposits histone H3.3 into nucleosomes in a DNA replication- and sequence-independent manner. As herpesvirus genomes enter the nucleus as naked DNA, we asked whether the HIRA chaperone complex affects herpesvirus infection. After infection of primary cells with HSV or CMV, or transient transfection with naked plasmid DNA, HIRA re-localizes to PML bodies, sites of cellular anti-viral activity. HIRA co-localizes with viral genomes, binds to incoming viral and plasmid DNAs and deposits histone H3.3 onto these. Anti-viral interferons (IFN) specifically induce HIRA/PML co-localization at PML nuclear bodies and HIRA recruitment to IFN target genes, although HIRA is not required for IFN-inducible expression of these genes. HIRA is, however, required for suppression of viral gene expression, virus replication and lytic infection and restricts murine CMV replication *in vivo*. We propose that the HIRA chaperone complex represses incoming naked viral DNAs through chromatinization as part of intrinsic cellular immunity.

## INTRODUCTION

The HIRA chaperone complex, comprised of HIRA/UBN1/CABIN1, collaborates with histone binding protein ASF1a to incorporate H3.3 into chromatin in a DNA replication-independent manner (1–3). The HIRA complex is targeted non-specifically to naked DNA and so is thought to deposit histone H3.3 through a so-called ‘gap-filling’ function (4). HIRA-mediated H3.3 deposition contributes to diverse biological functions. The HIRA protein is required for deposition of histone H3.3 at dynamic chromatin of active and poised genes and enhancers, polycomb target genes and sites of DNA damage repair (4–8). Accordingly, HIRA is required for gene activation in some contexts (9–11), but also chromatin silencing in others (12,13). HIRA’s role in H3.3 deposition is thought to underpin diverse physiological functions in metazoans, including sperm nucleus decondensation after fertilization (3), embryo development (5,14,15) and cell senescence and tumor suppression (16,17).

Cells encounter HIRA’s target—naked, histone-free DNA—in only special circumstances. Naked DNA is often a sign of microbial and viral infection (18). Consequently, foreign naked DNA is a trigger for activation of diverse cellular defense mechanisms, including anti-viral interferon (IFN) and cytokine responses that induce anti-viral genes and/or enhance the adaptive immune response. Specific DNA sensors detect microbial, histone-free DNAs. For example, in early stages of infection of fibroblasts with herpes simplex virus 1 (HSV-1), naked viral DNA enters the nucleus and is initially sensed by IFI16, which promotes activation of IFN and cytokine signaling via STING, NF-kB and the inflammasome (18). As well as leading to the activation of anti-viral IFNs and cytokines, naked viral DNAs also appear to directly activate intrinsic cellular immunity, a process leading to inhibition of viral gene expression and replication, potentially caused by heterochromatinization and silencing of the foreign viral DNA. On infection with several DNA viruses, this pathway is influenced by the activity of components of subnuclear structures, PML bodies, such as PML and Sp100 and, interestingly, another histone H3.3 chaperone, DAXX (19,20). IFI16 and a number of other host factors have also been proposed to contribute to virus heterochromatinization and silencing (21–23).

Given the role of the HIRA chaperone complex in nucleosome deposition at naked DNA, and the contribution of chromatinization of naked viral DNAs to anti-viral immunity, we set out to investigate whether HIRA participates in intrinsic anti-viral defense.

## MATERIALS AND METHODS

### Cell culture and IFN treatments

IMR90 cells were obtained from ATCC and were cultured in Dulbecco’s modified Eagle’s medium (DMEM) supplemented with 20% (v/v) FBS, 2 mM L-Glutamine and incubated at 37°C in a humidified 5% CO_2_ and 3% O_2_ atmosphere. HFFs (Diploid human foreskin fibroblasts, ECACC), U2OS cells (human osteosarcoma, ECACC) and HEK-293T cells (human embryonic kidney, ECACC) were propagated in DMEM supplemented with 10% v/v fetal calf serum (FCS, GE Health Care). BHK cells (baby hamster kidney, ECACC) were grown in Glasgow Modified Eagle’s Medium containing 10% newborn calf serum (NCS, GE Health Care) and 10% tryptose phosphate broth. HepaRG cells (Human hepatocytes, life Technologies) were grown in William’s Medium E containing 10% v/v FCS, 2 mM glutamine, 5 µg/ml insulin and 0.5 µM hydrocortisone. All cell growth media were supplemented with 100 U/ml penicillin and 100 µg/ml streptomycin and cells were incubated at 37°C in a humidified 5% CO_2_ and 3% O_2_ atmosphere. Lentivirus-transduced cells were maintained under continuous antibiotic selection, as appropriate. Recombinant IFNs were bought from PBL (Human IFN-β -11410–2) and Sigma (IFN-α-SRP4596 and IFN-γ-I3265). ELISA (PBL Assay Science 41410) was performed according to the manufacturer’s instructions.

### Viruses

HCMV strain AD169 (gen bank accession number FJ527563.1) was propagated in and titrated on HFFs as described previously (24). Virus was UV-inactivated using a Stratalinker 1800 (Stratagene) and three bursts of 800uJ (x100) and stored at −70°C. HSV-1 strain 17+ and its derivatives ICP0 null mutant dl1403 and *in*1318 (25) (gen bank accession number JN555585.1) were propagated in BHK cells, infected culture medium was harvested, cleared from cellular debris by centrifugation and titrated in U2OS cells, in which ICP0 is not required for efficient replication of HSV-1. HSV-1 mutant *in*1318 carries a temperature-sensitive lesion in ICP4, a deletion of the ICP0 gene and a mutation within VP16 that inactivates its ability to stimulate IE gene expression and was propagated at 31°C as described previously (26). Viruses *in*1863 and dl1403/CMV/lacZ are wild-type (WT) and ICP0-null (respectively) derivatives of HSV-1 17+ containing the lacZ gene under control of the HCMV promoter/enhancer inserted into the tk gene (27).

### *In vitro* virus infections

For co-localization analysis, cells were seeded into 6-well dishes (with or without coverslips) at 5 × 10^5^ cells per well, infected the following day with in1318 or UV-HCMV at multiplicity of infection (MOI) 5 and incubated for 6 days. For immunofluorescence, cells were seeded at 1 × 10^5^ cells per well into 24-well dishes on coverslips, infected the next day with HSV-1 mutant dl1403 at MOI 0.4 and fixed and stained 24 h post-infection. For plaque assays, cells were seeded as above and infected the following day with appropriate sequential 3-fold dilutions of WT HSV-1 variant *in*1863 and ICP0-null HSV-1 mutant *dl*1403/CMV/lacZ. After virus adsorption, cells were overlaid with medium containing 1% human serum for 24 h and stained for β-galactosidase-positive plaques as described (28). For virus yield, cells were seeded as above and infected the following day at MOI 0.01. At indicated time points post infection, supernatant was harvested and virus titre determined by plaque formation assay on U2OS cells as described previously (29). For western blot analysis, cells were seeded into 24-well dishes at 1 × 10^5^ cells per well, infected at indicated MOIs and harvested at indicated times post infection. For ChIP assays, cells were seeded at 3 × 10^6^ cells per 15 cm dish and infected the following day with UV HCMV or in1318 at MOI 20. Cells were incubated for 24 h at 37°C (UV HCMV) or at the non-permissive temperature of 38.5°C (*in*1318).

### ChIP

Native ChIP was performed for HIRA and HA-H3.3 as described previously (17). Antibodies used for ChIP were: cocktail of mouse mAbs to HIRA (approximately equimolar mixture of WC15, WC19, WC117, WC 119 (30)), HA (Millipore 05904) (for anti-HA-H3.3 ChIP). Mouse mAb to HA tag (Covance, MMS-101R) was used as species/class-matched negative control Abs. Primer sequences are provided in Supplementary Table S1.

### Generation and production of lentivirus vectors

Hairpins to HIRA and luciferase, and lentivirus vectors expressing an HA-tagged version of human histone H3.3 have been described previously (17).

### qRT-PCR

Total RNA was prepared using the RNeasy kit (Qiagen catalog no. 74104), according to the manufacturer’s instructions. Quantitative reverse transcription-polymerase chain reaction (qRT-PCR) was performed using the Dynamo SYBR green kit according to the manufacturer's instructions. β Actin was used as housekeeping control.

### Western blot

Western blotting was performed as described previously (17). The following primary antibodies were used for western blot: mouse mAb to HIRA (WC119 (30)), actin (A1978; Sigma), MAb anti-HCMV-IE1 2470–5604 (AbD Serotec), HSV1-ICP8 MAb [11E2] ab20194 (Abcam), anti-UL42 MAb Z1F11 (31), anti-actin rabbit serum A5060 (Sigma-Aldrich), SV40 T Ag (Santa Cruz sc-147), p53 (Santa Cruz sc-126), pRb (Cell Signalling Technology 9309S).

### Immunofluorescence

Two-color indirect immunofluorescence assays were performed as described previously (32). Primary antibodies used in this study were: cocktail of mouse mAbs to HIRA (approximately equimolar mixture of WC15, WC19, WC117, WC 119 (30)), histone H3 (39 163, 1:1000; Active Motif), promyelocytic leukemia body (anti-PML) (sc-966 and sc5621; Santa Cruz). The secondary antibodies used were Alexa Fluor 488 conjugated goat anti-mouse (life technologies, A-11001) and Alexa Fluor 555 donkey anti-rabbit (life technologies, A-31572). For confocal microscopy samples were examined using a Zeiss LSM 510 confocal microscope with 488 and 543 nm laser lines, scanning each channel separately under image capture conditions that eliminated channel overlap. The images were exported as TIFF files and processed using Adobe Photoshop and Adobe Illustrator.

### *In vivo* experiments

Experiments were performed under the UK Home Office (project license number PPL 70/8354) guidelines in line with the EU Directive 2010 and local Ethical Review process (University of Glasgow). The University of Glasgow ethics committee reviewed and approved the document and the experiments conducted for this study. Murine cytomegalovirus (MCMV) infections performed at Cardiff University were under the UK Home Office license PPL 30/2969. All lines were maintained on a C57Bl/6J background. Mice were infected with 3 × 10^4^ PFU salivary gland-propagated Smith strain MCMV via the intra-peritoneal (i.p) route of administration. Virus stocks and virus-infected tissue homogenates were titred by plaque assay using 3T3 fibroblasts. The alleles used for this study were as follows: *Hira*^fl/fl^, *CAGG-Cre-ER* (17). Genotyping was carried out by Transnetyx (www.transnetyx.com). Recombination by *CAGG-Cre-ER* was induced with a single i.p. injection of tamoxifen (80 mg/kg) made up in 10% ethanol and 90% corn oil, daily for 5 days when mice were 6–8 weeks of age. WT mice were *CAGG-Cre-ER* (*n* = 8) and HIRA knockouts were *Hira*^fl/fl^, *CAGG-Cre-ER* (*n* = 18).

### Statistical methods

For pairs of transfections in which experiments were repeated three times, we performed the exact Cochran-Mantel-Haenszel (CMH) test to determine whether or not, adjusting for experiment, there was a higher proportion of cells with HIRA localized to PML bodies in one transfection relative to the other. For every individual experiment a minimum of 100 cells were counted. The CMH test’s assumption of homogeneity of odds ratios was checked using Woolf’s test; in cases where this assumption was violated, Fisher’s exact test was performed on each individual experiment and the largest *P*-value was selected. All reported *P*-values were adjusted using Bonferroni correction.

For secreted IFN-β comparison a Mann–Whitney test was performed. This produced a borderline-significant difference between the two groups (Mock and pcDNA3), with a *P*-value of 0.04953. For affect of knockdown in recruitment of H3 to PML bodies, the data were analyzed using a *t*-test. Both comparisons were found to be significant at the 5% level (Bonferroni-adjusted *P*-value for shCntrl versus sh H1 was 0.0136; Bonferroni-adjusted *P*-value for shCntrl versus sh H2 was 0.0499).

### Data analysis

Reads were trimmed using Trim Galore (v0.3.0) (http://www.bioinformatics.babraham.ac.uk/projects/trim_galore/) and quality assessed using FastQC (v0.10.0) (http://www.bioinformatics.bbsrc.ac.uk/projects/fastqc/). For RNA-seq analysis paired-end reads are aligned to the human genome (hg19) using a splicing-aware aligner (tophat2) (33). Duplicate reads were identified using the picard tools (1.98) script mark duplicates (http://picard.sourceforge.net.). Only non-duplicate reads were retained. See Supplementary Table S1. Reference splice junctions are provided by a reference transcriptome (Ensembl build 73), and novel splicing junctions are determined by detecting reads that span exons that are not in the reference annotation. Aligned reads are processed to assemble transcript isoforms, and abundance is estimated using the maximum likelihood estimate function (cuffdiff) from which differential expression and splicing can be derived (34). Genes of significantly changing expression were defined as FDR corrected *P*-value < 0.05. Only ensembl 73 genes of status ‘known’ and biotype ‘coding’ were used for downstream analysis.

To generate heatmaps, first, significantly changing genes between shLuc 0 and 6 h, and 6 and 24 h of status ‘known’ and biotype ‘coding’ were identified. For each gene the FPKM value was calculated based on aligned reads, using Cufflinks (34). *Z*-scores were generated from FPKMs. Hierarchical clustering was performed using the R library heatmap.2 and the distfun = ‘pearson’ and hclustfun = ‘average’. Principal component analysis (PCA) was performed using the FPKM values of all ensembl 73 genes of status ‘known’ and biotype ‘coding’. To calculate the proportion of genes with concordant expression profiles between IFN-β shLuc and IFN-β shHIRA, first all genes which changed significantly (5% FDR) in expression between shLuc IFN-β 0 and 6 h or between shLuc IFN-β 6 and 24 h were identified. Next the genes in the gene set were classified into four expression profiles—upregulated/upregulated, upregulated/downregulated, downregulated/downregulated and downregulated/upregulated, dependent on the direction of change in expression from 0 to 6 h and 6 to 24 h respectively. Next, for the same gene set, the expression profiles were similarly determined using the shHIRA IFN-β datasets. The proportion of genes whereby the profile was concordant between the IFN-β shLuc and IFN-β shHIRA datasets was calulated.

For analysis ChIP-seq single-end reads are aligned to the human genome (hg19) using the Bowtie2 alignment software (35). Duplicate reads were identified using the picard tools (1.98) script mark duplicates (http://picard.sourceforge.net.). Only unique reads mapping to a single location were retained (see Supplementary Table S2). HIRA binding sites were determined using the USeq package (v8.6.0) (36). For USeq the window size was 200 bp, the extension size was 150 bp. To create the enriched regions, the following parameters were used: -i 2,4 -s 13,1. Input DNA was used as the normalization control. The ChIP-seq signal for any given window was calculated as the total number of fractional reads within a window, divided by the window length, with the product divided by the total number of reads in the dataset divided by 1 million. To normalize the window the ChIP-seq signal of the treatment was divided by the control signal. Venn diagrams and associated empirical *P*-values were generated using the USeq (v7.1.2) tool IntersectLists (36). The −t value used was 22 008, as the total number of genes of status ‘known’ and biotype ‘coding’ in ensembl genes 73. The number of iterations used was 1000.

## RESULTS

### HIRA chromatinizes incoming viral and other foreign DNAs

**Figure 1.**
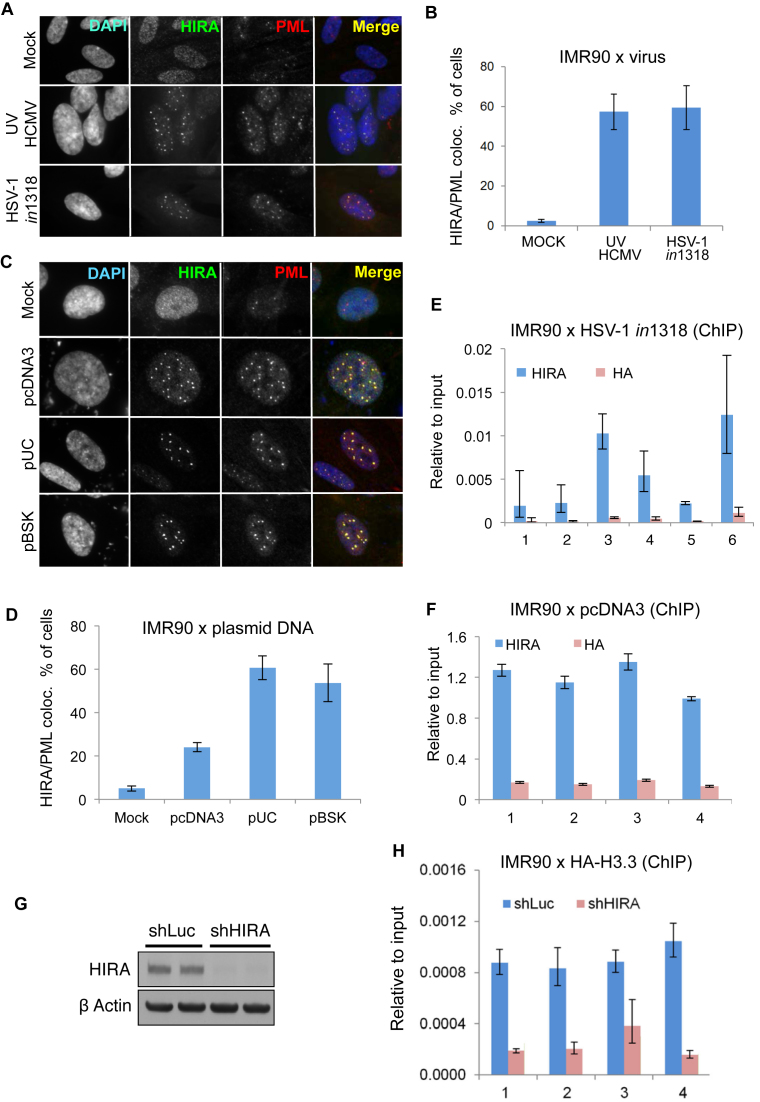
HIRA chromatinizes incoming viral and other foreign DNAs. (**A**–**D**) IMR90 cells fluorescently stained with antibodies to HIRA and PML. (A) Cells were infected at MOI 5 with UV-inactivated human CMV strain AD169 (UVHCMV) or a replication deficient HSV-1 variant (in1318). Cells were fixed and stained at 24 h post-infection. (B) Quantitation of cells with HIRA localized to PML bodies from (A). Data are mean ± Standard Error Mean (SEM) from three independent experiments, *P* < 0.05 comparing mock versus UVHCMV and in1318. (C) Cells transiently transfected with the indicated plasmid DNAs. (D) Quantitation of cells with HIRA localized to PML bodies from (C). Data are mean ± SEM from three independent experiments, *P* < 0.05 comparing mock versus plasmid transfections. (**E** and **F**) IMR90 cells were infected with a replication deficient HSV-1 variant (*in*1318) at MOI20 (E) or transfected with pcDNA3 (F). Lysates were harvested at 24 h post-infection or transfection and DNA binding measured by anti-HIRA ChIP assay (using anti-HA as a negative control) using PCR primers binding different regions throughout the input DNA (Supplementary Table S3). For (E) data is mean ± SEM. Error bars are from technical replicates. Experiment was done three independent times with similar results. Numbers 1–6 represents six distinct regions on HSV-1 variant (*in*1318) genome (Supplementary Table S3). For (F) data are mean ± SEM from three independent experiments. *P* < 0.05 for all regions compared to control IgG (HA). Numbers 1–4 represents four distinct regions on pcDNA3 genome (Supplementary Table S3). (**G**) IMR90 cells were infected with lentiviruses encoding shRNAs against HIRA (shHIRA) or control (shLuc) and immunoblotted with indicated antibodies. (**H**) Cells from (G) were transfected with pcDNA3 and infected with purified HA-H3.3 lentivirus. Lysates were harvested at 24 h post-transfection and DNA binding measured by ChIP assay using PCR primers binding different regions throughout the input DNA. Data are mean ± SEM from three independent experiments. Numbers 1–4 represents four distinct regions on pcDNA3 genome (Supplementary Table S3). *P* < 0.05 for all regions comparing shLuc to shHIRA.

First, we tested whether HIRA would co-localize to and interact with histone-free herpesvirus genomes. To prevent viral proteins from counteracting the cellular anti-viral response, cells were infected with UV-inactivated WT human cytomegalovirus strain AD169 (UVHCMV) or an HSV type 1 strain expressing a lacZ marker gene (*in*1318) and harboring mutations in the viral transactivator proteins ICP0, ICP4 and VP16 which normally counteract cellular repressors. These viral genomes rapidly enter into a quiescent, transcriptionally repressed state immediately upon infection. In human fetal lung fibroblasts (IMR90), HIRA localized to PML bodies in response to infection with UVHCMV or HSV *in*1318 (Figure 1A and B). Consistent with re-localization of HIRA to PML bodies being, directly or indirectly, a response to naked viral DNA, transient transfection with purified naked plasmid DNAs, pcDNA3, pUC or pBSK, also triggered re-localization of HIRA to PML bodies (Figure 1C and D). Moreover, in virus-infected or plasmid-transfected cells, HIRA directly or indirectly bound to the foreign DNA, as measured by ChIP assay (Figure 1E and F; Supplementary Figure S1A and B). HIRA’s substrate, histone H3.3, was deposited onto foreign plasmid DNA and this depended on HIRA (Figure 1G and H). In sum, we conclude that in cells harboring foreign transfected plasmid or infected viral DNAs, HIRA is recruited to PML bodies, proposed sites of anti-viral activity. Moreover, HIRA binds to incoming viral and other foreign DNAs and promotes their chromatinization via deposition of histone H3.3.

### Histone chaperone HIRA responds to interferon

**Figure 2.**
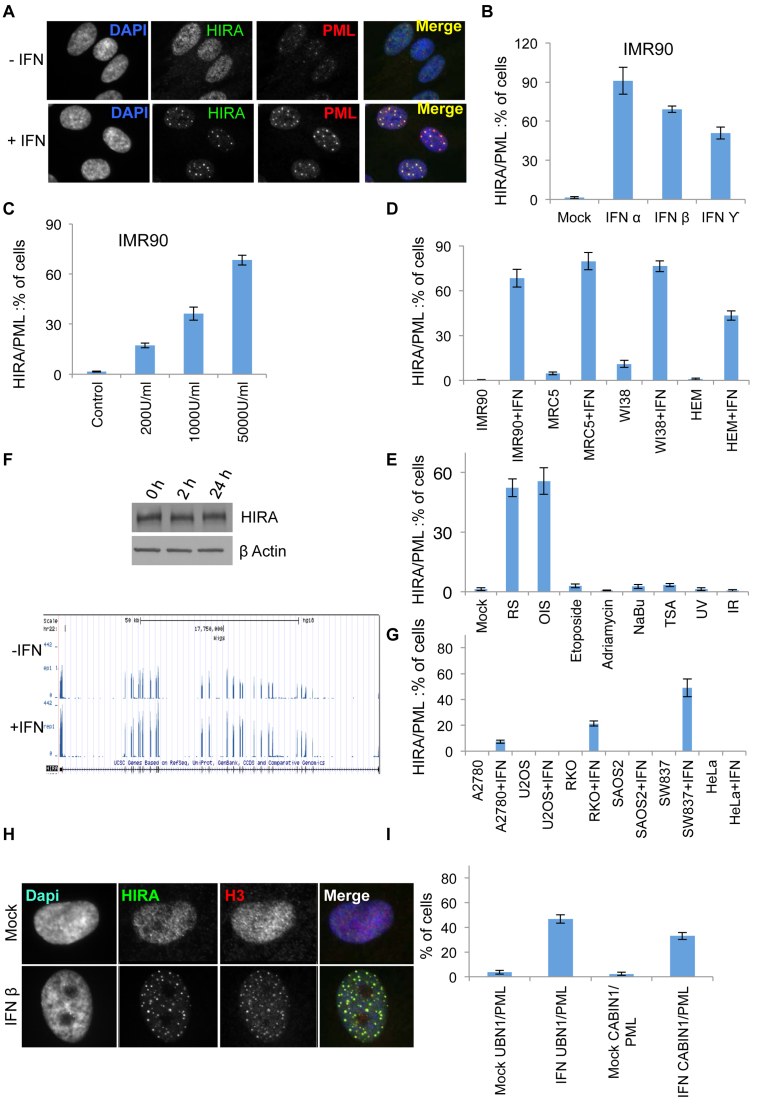
Histone chaperone HIRA responds to IFN. (**A**) IMR90 cells fluorescently stained with antibodies to HIRA and PML 24 h post-treatment with 2000U/ml of IFN-β. (**B**) Quantitation of cells with HIRA localized to PML bodies from (A) and with cells treated with 2000U/ml each of IFN-α and IFN-γ. Data are mean ± SEM from three independent experiments, *P* < 0.05 comparing mock versus IFN-treated cells. (**C**) Quantitation of cells with HIRA localized to PML bodies in cells treated with increasing dose of IFN-β as indicated. Data are mean ± SEM from three independent experiments, *P* < 0.05 as compared to mock for all three doses. (**D**) Quantitation of cells with HIRA localized to PML bodies in primary cells (IMR90 fibroblasts, MRC5 fibroblasts, WI38 fibroblasts, human epidermal melanocytes (HEM)) treated with and without 2000U/ml of IFN-β. Data are mean ± SEM from three independent experiments, *P* < 0.05 as compared to its mock treated for each independent cell line. (**E**) Quantitation of cells with HIRA localized to PML bodies in cells with indicated triggers and replicative (RS) and oncogene-induced senescent (OIS) cells. Data are mean ± SEM from three independent experiments, *P* < 0.05 as compared to its mock treated for RS and OIS, p is non-significant for all other conditions. (**F**) Left panel: IMR90 cells treated with 2000U/ml of IFN-β and western blotted with indicated antibodies. Right Panel: genome browser representation of mRNA of HIRA in IMR90 cells treated with and without 2000U/ml of IFN-β. (**G**) Quantitation of cells with HIRA localized to PML bodies in cancer cell lines (A2780, U2OS, RKO, SAOS2, SW837 and HeLa) treated with and without 2000U/ml of IFN-β. Data are mean ± SEM from three independent experiments, *P* < 0.05 for A2780, RKO and SW837 cells as compared to their mock treated cells. p is non-significant for all other cell lines. (**H**) Cells from (A) were fluorescently labeled with antibodies to HIRA and histone H3. (**I**) Quantitation of cells with UBN1 and CABIN1 localized to PML bodies in IMR90 cells treated with and without 2000U/ml of IFN-β. Data are mean ± SEM from three independent experiments, *P* < 0.05 as compared to mock.

Virus infection is a potent activator of the cellular anti-viral IFN response (37). Transient transfection with plasmid DNA has also been reported to cause secretion of IFN (38). Therefore, we reasoned that re-localization of HIRA in plasmid-transfected and virus-infected cells might be caused, at least in part, by secreted IFN. Indeed, conditioned medium from plasmid-transfected cells contained increased IFN-β (Supplementary Figure S2A), and conditioned medium and purified IFNs were dose-dependent inducers of HIRA’s localization to PML bodies in primary cells, including in several fibroblast strains and melanocytes (Figure 2A–D and Supplementary Figure S2B and C). We and others previously showed that HIRA progressively accumulates in PML bodies of primary fibroblasts with increasing number of population doublings, and in cells nearing or at senescence most cells contained HIRA in PML bodies as reported previously (16,17,39). However, a panel of other diverse cell stresses (etoposide, adriamycin, UV light and ionizing radiation) failed to recruit HIRA to PML bodies (Figure 2E). Moreover, although IFNs have been reported to induce cellular senescence (40–43), HIRA’s localization to PML bodies in response to IFN was not a consequence of senescence, as this phenomenon also occurred in IMR90 cells stably expressing SV40 T antigen to disable the cell senescence program (Supplementary Figure S2D–F). IFN-stimulated recruitment of HIRA to PML bodies was not a consequence of increased expression of HIRA, since there was only a modest change in HIRA expression at the RNA level and no detectable change at the protein level (Figure 2F). Interestingly, HIRA’s localization to PML bodies was impaired in several, but not all, cancer cell lines (Figure 2G). As in senescent cells, localization of HIRA to PML bodies was accompanied by similar re-localization of histone H3 and other members of the chaperone complex, UBN1 and CABIN1 (Figure 2H and I) (16,17,44,45). We conclude that in primary cells anti-viral IFNs can trigger re-localization of the HIRA complex to PML bodies, further implicating this chaperone complex in the cellular anti-viral response.

### HIRA is not required for activation of IFN target genes

**Figure 3.**
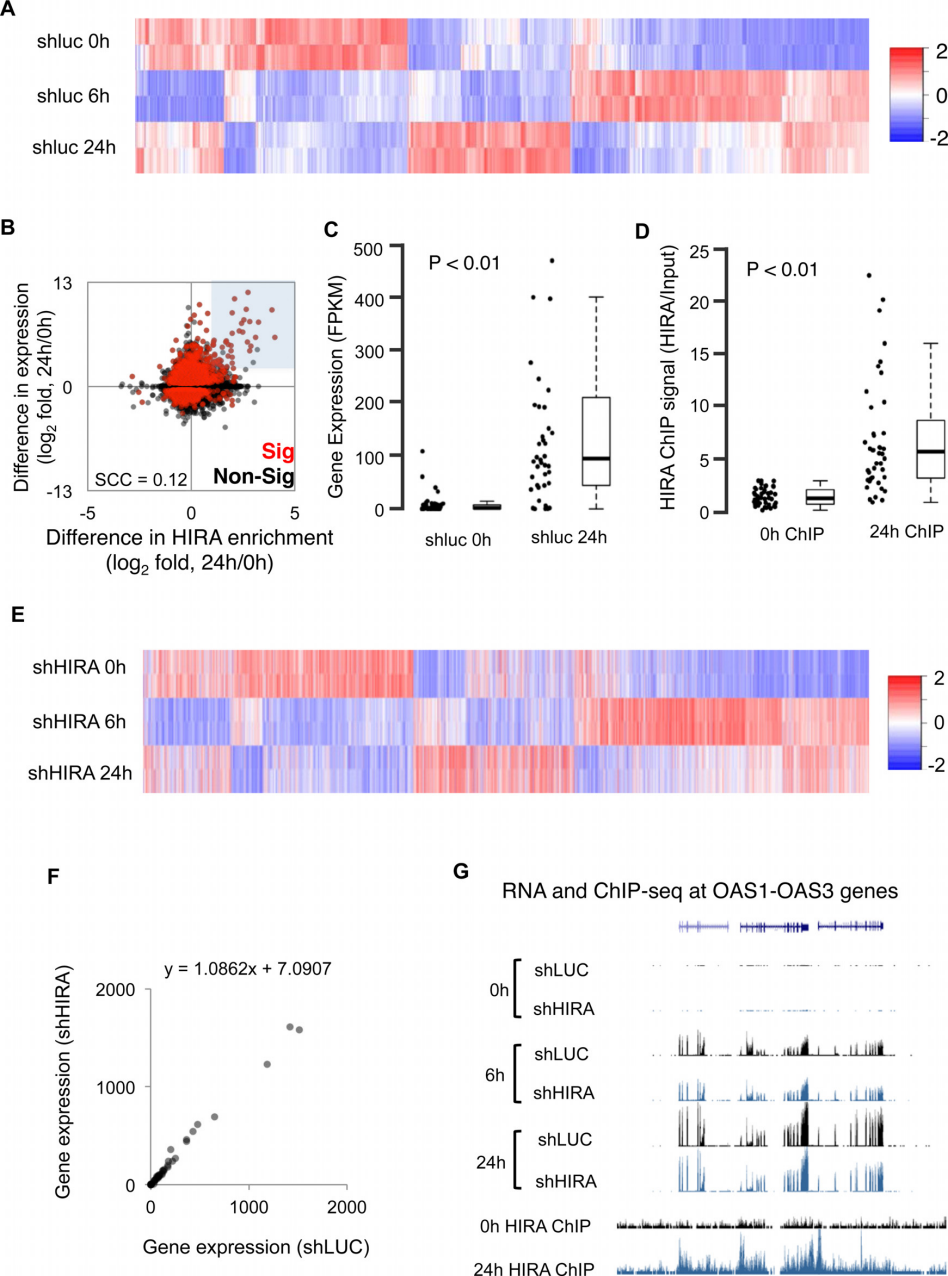
HIRA is not required for activation of IFN target genes. (**A**–**G**) IMR90 cells were stably infected with lentivirus-encoded shRNAs to HIRA (shHIRA) or control (shLuc). Cells were then treated with IFN-β either for 6 h or for 24 h as indicated in the legend. (A) Column clustered heatmap of all differentially expressed genes (FDR ≤ 5%) between shLuc 0 and 6 h and/or between shLuc 6 and 24 h. Genes are given by column and samples by row. The color intensity represents column *Z*-score (based on FPKM), where red indicates more highly expressed, and blue more lowly expressed genes. (B) Scatter plot comparing the change in promoter (TSS ± 1 kb) HIRA enrichment with change in gene expression after IFN-β treatment for 24 h. Genes that change in expression significantly (FDR ≤ 5%) between shLuc 0 and 24 h are in red. All other genes are in black. HIRA ChIP-seq signals have been normalized to input control. The gray highlighted section shows genes with an expression fold ≥ 2.5 and HIRA fold ≥ 1 (also in Supplementary Figure S3D). (C) Box and jitter plots of shLuc 0 and 24 h gene expression (mean FPKM, *n* = 2) for the genes within the highlighted section of panel B. The *P*-value was calculated using a Wilcoxon rank-sum test, comparing 0–24 h. The bottom and top of the boxes correspond to the 25th and _^75th^_ percentiles respectively, and the internal band is the median. The plot whiskers correspond to the most extreme value within 1.5 × interquartile range. (D) As 3C, though measuring HIRA enrichment by ChIP-seq at 0 and 24 h. HIRA ChIP-seq signals have been normalized to input control. (E) Heatmap of the genes in A, ordered as 3A. Samples are HIRA knockdown using an shRNA hairpins (shHIRA). Genes are given by column and samples by row. The color intensity represents column *Z*-score (based on FPKM), where red indicates more highly expressed, and blue more lowly expressed genes. The experiment was done in parallel with shLuc cells in (A); however to simply heatmap, only results from the knockdown cells are shown (shHIRA). Indicated time points refer to hours post IFN-β treatment. (F) Scatter plot of the genes within the highlighted section of (B), comparing shLuc to shHIRA expression. Values are mean FPKMs (*n* = 2). The straight-line equation derived from a linear regression is given for both comparisons. Slope of the line indicates no difference between shLuc and shHIRA. (G) Representative UCSC plot of the OAS1–3 gene cluster, showing expression by RNA-seq (top six tracks) and HIRA enrichment by ChIP-seq (bottom two tracks). The Y-axis represents library normalized read count. For the gene tracks, introns are given as horizontal lines and exons as vertical boxes.

PML bodies have been proposed to facilitate routing of histones and histone chaperones from the nucleoplasm to the chromatin (17,39,46,47). Therefore, we reasoned that HIRA’s recruitment to PML bodies in IFN-treated, virus-infected and plasmid-transfected cells might, in part, reflect its recruitment to IFN-inducible genes and a contribution to their inducible expression. As shown previously (37), treatment of primary human fibroblasts with IFN resulted in widespread time-dependent increases and decreases in gene expression (Figure 3A, Supplementary Figure S3A and Table S1). Although canonical IFN target genes were a small proportion of the total regulated genes, the set of regulated genes included more than 50% of all such IFN target genes (Supplementary Figure S3B). To test whether HIRA might be involved in control of IFN-regulated genes, we performed ChIP-seq to determine the distribution of HIRA across the genome in untreated control cells and cells treated with IFN for 24 h (Supplementary Table S2). Initial analysis of selected IFN target genes, such as IFITM1, confirmed that increased expression of these genes was often associated with increased binding of HIRA across the promoter and exons (Supplementary Figure S3C). However, across all genes there was a relatively poor correlation between change in expression and change in HIRA binding after IFN treatment (Spearman Correlation Coefficient = 0.12; Figure 3B). Despite this relatively poor global correlation, a subset of genes showed a marked positive correlation between fold change in expression and fold change in HIRA binding (shaded in Figure 3B and Supplementary Figure S3D (PCC = 0.62)). At these genes, a marked increase in HIRA binding was often accompanied by a marked increase in expression, often from a very low level in untreated cells (Figure 3C and D). These genes were more than 30-fold enriched for known up regulated IFN-β target genes (Supplementary Figure S3E).

To test whether upregulation of these genes depends on HIRA, we generated primary human fibroblasts lacking HIRA via lentivirus-encoded shRNA-mediated knockdown (Figure 1G and Supplementary Figure S3F), and treated these cells with and without IFN. As shown previously (17), HIRA knock down blocked overt IFN-induced recruitment of histone H3 to PML bodies (Supplementary Figure S3G and H) (although with any partially diffuse nuclear stain, some co-localization is inevitable and functional significance of this cannot be excluded). However, to our surprise, RNA-seq analysis showed that knock down of HIRA had only a very modest effect on global IFN-induced changes in gene expression (Figure 3E, compare to Figure 3A). PCA showed that control cells and HIRA deficient cells tended to cluster together (Supplementary Figure S3I), and ∼80% of IFN-regulated genes showed concordant expression between control cells and HIRA deficient cells (Supplementary Figure S3J). Most strikingly, knock down of HIRA had no significant effect even on expression of those genes that bound HIRA and increased expression after IFN treatment (Figure 3F). For example, although IFN target genes OAS1–3 bound HIRA after IFN treatment, knock down of HIRA had no effect on their IFN inducible expression (Figure 3G). Based on these data, we conclude that, while HIRA is recruited following IFN treatment to many of the most upregulated IFN responsive genes, neither expression of HIRA nor recruitment of histone H3 to PML bodies is generally required for activation of these genes.

### HIRA is required for efficient suppression of viral infection

**Figure 4.**
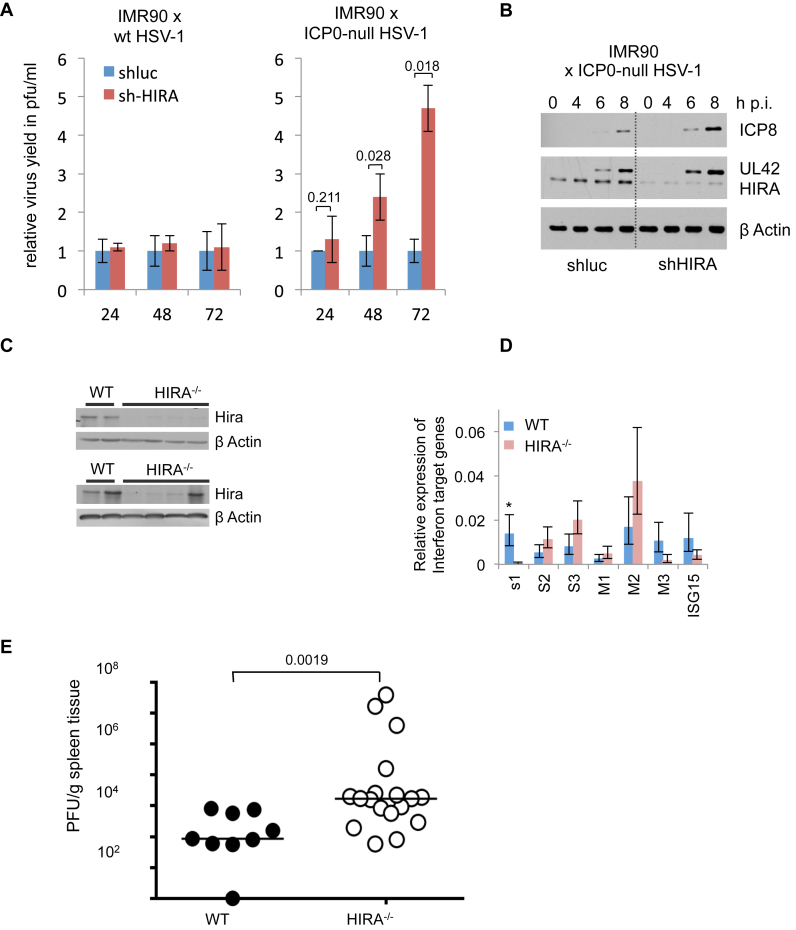
HIRA contributes to efficient suppression of viral infection. (**A**) Virus yield from HIRA-depleted and control IMR90 cells infected at MOI 0.01 with ICP0-null HSV-1 mutant *dl*1403 CMV lacZ or wt HSV-1 variant *in*1863. Supernatant was harvested at indicated times post infection (h p.i.) and virus titres determined by plaque assay. Data are mean +/- SD (error bars) (n = 3 biological repeats) with indicated *P* values. (**B**) HIRA-depleted and control IMR90 cells infected with ICP0-null HSV-1 mutant *dl*1403 at MOI 2.0. Lysates were harvested and processed at indicated time points post infection (h p.i.) (**C**–**E**) Control (*CAGG-Cre-ER*, WT, +tamoxifen) or Hira-deficient (*CAGG-Cre-ER, Hirafl/fl*, +tamoxifen) mice were infected with MCMV and spleen harvested 4 days later. Each spleen was divided into three pieces for downstream analysis. (C) Western blot analysis of WT or *HIRA^−/−^* animals showing knock out of Hira. Shown are representative western blot results from two different gels with 4 WT and 8 *HIRA^−/-^*mice. (D) mRNA abundance of IFN-β target genes by qRT-PCR. Bar chart displays mean of each IFN-β target gene mRNA abundance in WT mice compared to *HIRA^−/-^*mice, normalized to β*-*actin as housekeeping control. Data are mean ± SEM (error bars) (*n* = 4 for WT mice and *n* = 8 for *HIRA^−/-^*mice). *P < 0.05 (OAS1). *P* > 0.05 for the other six genes (OAS2, OAS3, IFITM1, IFITM2, IFITM3 and ISG15). (E) Plaque forming units measured per gram of tissue of WT or *HIRA^−/−^* mice. *P*-value assessed by Mann–Whitney-U test (*n* = 9 for WT mice and *n* = 18 for *HIRA^−/-^*mice).

These data implicate HIRA in the cellular response to virus infection and IFN, but do not define a specific anti-viral function for HIRA. Given that HIRA binds to and chromatinizes viral templates (Figure 1), we speculated that HIRA might directly impact viral DNA replication. To directly test a role for HIRA in control of viral replication, we infected control and HIRA-depleted IMR90 cells with the WT HSV-1 and ICP0-null HSV1 virus *dl*1403. We observed an increase in virus yield from the infected cells in absence of HIRA in infection with HSV lacking the viral transcriptional activator ICP0 (Figure 4A). Furthermore, knock down of HIRA also led to an earlier onset of the viral gene expression program in absence of ICP0 (Figure 4B). Finally, we asked whether HIRA suppressed viral replication during herpesvirus infection *in vivo*. For this, we employed conditional Hira knock out mice (*CAGG-Cre-ER, Hirafl/fl*) in which tamoxifen-inducible inactivation of Hira is directed by a ubiquitously expressed Cre-ER fusion protein under control of a *CAGG* promoter (17). Control (WT, +tamoxifen) or HIRA-deficient (*Hirafl/fl*, +tamoxifen) mice were infected with the β−herpesvirus MCMV and virus load in the spleen was quantified 4 days later. Western blotting confirmed efficient inactivation of Hira in the majority of spleens (Figure 4C). Consistent with the *in vitro* data this had no consistent effect on expression of IFN target genes (Figure 4D). However, *Hira^−/−^*mice exhibited a substantially increased viral load in spleen and liver (Figure 4E and Supplementary Figure S4), reflecting impaired control of acute MCMV replication. Based on these data, we conclude that the Hira chaperone complex participates in anti-viral intrinsic immunity.

## DISCUSSION

Here we demonstrate that the HIRA histone chaperone complex is involved in chromatinization of incoming viral DNAs and participates in cellular anti-viral intrinsic immunity. Several lines of evidence support this view. First, HIRA relocalizes to PML nuclear bodies in response to incoming viral and foreign naked DNAs. PML bodies have been previously linked to immunological responses to viral infection (19,20). Second, HIRA binds to incoming viral and other foreign DNAs and promotes their chromatinization by depositing histone H3.3. Third, HIRA responds to anti-viral IFNs, by localizing to PML nuclear bodies and to many upregulated IFN responsive genes. Fourth, while HIRA is not required for IFN inducible gene expression, we observed an increase in virus yield from infected cells in absence of HIRA, after infection with HSV lacking the viral transcriptional activator ICP0. Fifth, viral gene expression is more efficient in HIRA-depleted cells than control cells, suggesting an important role for HIRA in cellular anti virus response. Finally, mice lacking HIRA have a significantly higher viral load than control mice, suggesting an essential requirement of HIRA in virus silencing *in vivo*. Together, these lines of evidence indicate that HIRA may contribute to viral gene silencing and thus play a key role in cellular anti-viral intrinsic immunity.

In apparent contrast to our findings, Placek *et al.* previously reported that HIRA activates HSV-1 gene expression and promotes genome replication, both also linked to H3.3 deposition (10). Interestingly, however, Placek *et al.* performed their studies in HeLa cells. We found that HeLa cells, and many other transformed human cell lines, fail to recruit HIRA to PML bodies in response to IFN. This suggests that the anti-viral activity of HIRA may be cell type specific, and/or that the anti-viral properties of HIRA are inhibited or counteracted in some cancer cells.

We propose that HIRA’s participation in intrinsic anti-viral immunity is linked to its previously demonstrated ability to non-specifically deposit nucleosomes onto naked DNA via a gap-filling mechanism (4). Other members of HIRA complex, UBN1 and CABIN1 also bind naked DNA in a non-specific manner (4). In addition, the HIRA complex likely confers other anti-viral mechanisms, as UBN1 has previously been reported to restrict the productive cycle of another human herpesvirus, EBV, through binding to the EBV EB1 protein (48,49). While the alternative H3.3 chaperone complex ATRX/DAXX is also involved in anti-viral intrinsic immunity (28,50–52), ATRX and DAXX do not bind to naked DNA under the same conditions as HIRA, UBN1 and CABIN1 (4), suggesting that their anti-viral mechanism might be distinct to HIRA. Although histone H3.3 is often associated with active transcription (53,54), the HIRA complex and its orthologs, together with histone H3.3, are also implicated in creating a repressive chromatin environment (12,13,55,56), likely underlying its anti-viral function.

In sum, these results, in cell culture and a mouse model, demonstrate a role of histone chaperone HIRA in sensing incoming foreign DNAs, suppression of viral gene expression and productive viral infection both *in vitro* and *in vivo*. We propose that HIRA’s ‘gap filling’ mode of DNA chromatinization is targeted to both the host genome for control of epigenome function and also to foreign DNAs for suppression of pathogenic infection.

## DATA AVAILABILITY

Sequences have been deposited in GEO (https://www.ncbi.nlm.nih.gov/geo/) under accession number GSE74863.

## Supplementary Material

Supplementary Data
