# Supplementary material for: Histone chaperone HIRA deposits histone H3.3 onto foreign viral DNA and contributes to anti-viral intrinsic immunity
Source: Nucleic Acids Res. 2017 Sep 13;45(20):11673–83. doi: 10.1093/nar/gkx771 (PMC5691367; doi:10.1093/nar/gkx771)
Supplement: Supplementary Data [file gkx771_supp.pdf]

Figure S1

(A and B) IMR90 cells were transfected with plasmids pUC and pBSK, as indicated. Lysates were harvested at 24h post infection or transfection and DNA binding measured by anti-HIRA ChIP assay (anti-HA as negative control) using primers binding different regions throughout the input DNA. Data are mean +/- SD (error bars) (n=3 biological repeats). $p<0.05$ for all regions as compared to control IgG (HA). PCR primer pairs in Supplementary Table S3.

Figure S2

(A) IMR90 cells were transfected with plasmid pcDNA3. 24 hours post-transfection level of secreted IFN-β was measured using ELISA. Data are mean +/- SD (error bars) (n=3 biological repeats). $p<0.05$ comparing mock vs pcDNA3 transfection. **(B)** IMR90 cells were transfected with pcDNA3, media was harvested 24 hours post-transfection and applied to un-transfected cells for another 24 hours. Cells were fluorescent stained with antibodies to HIRA and PML. **(C)** Quantitation of cells with HIRA localized to PML bodies from (B). Data are mean +/- SEM from three independent experiments, $p<0.05$ as compared to mock treated. **(D)** IMR90 cells were infected with lentivirus encoding SV40 T antigen and western blotted with indicated antibodies. Mock-uninfected lysate was used as control. **(E)** Cells from D were treated with 2000U/ml of IFN-β and fluorescent stained with antibodies to HIRA and PML. **(F)** Quantitation of cells with HIRA localized to PML bodies from (E). Data are mean +/- SEM from three independent experiments, $p<0.05$ as compared to mock treated.

Figure S3

(A) Venn diagram showing the overlap between genes that change in expression (FDR <= 5%) between 0h-6h of IFN-b (red circle) and between 6h-24h of IFN-b (blue circle) in shLuc IMR90 cells. **(B)** As B, however also showing the overlap with canonical IFN-β target genes (green circle) (https://interferome-v1.erc.monash.edu.au/). The empirical p value for the overlap between 0h-6h IFN-b and IFN-β target genes was <0.001, and between 6h-24h IFN-b and IFN-β target genes was <0.001 **(C)** Representative UCSC plot of the IFITM1 gene. Showing expression by RNA-seq (top four tracks) and HIRA enrichment by ChIP-seq (bottom two tracks). The Y-axis represents library normalized read count for each track. For gene track, intron is shown as thin horizontal line, exons as thick lines (5' and 3' UTR intermediate thickness). 0hr (untreated) and 24hr post-treatment with IFN-β. **(D)** Scatter plot comparing the change in promoter (TSS+/-1kb) HIRA enrichment with change in gene expression after IFN-β treatment for 24h. HIRA ChIP-seq signals have been normalised to input control. Showing only those genes from the grey highlighted section in Figure 3B (expression fold >= 2.5 and HIRA fold >=1). **(E)** Venn diagram showing the overlap between canonically up regulated IFN-β target genes (red circle, IFN-β Up) and the genes within the highlighted section of Figure 3B (blue circle, HIRA+, Exp+). The 14 genes in the overlap are ISG15, IFI44, IFI6, IFIT2, IFIT3, IFIT1, IFIT5, OAS1, OAS2, OASL, BST2, NMI, STAT1, MX2. The empirical p value for the overlap is <0.001 the fold enrichment is 33.17. **(F)** Representative UCSC plot of the HIRA gene. Showing gene expression by RNA-seq in control (shLuc) and HIRA knockdown (shHIRA). The Y-axis represents library normalized read count for each track. For gene track, introns are shown as thin horizontal line, exons as thick lines. **(G)** IMR90 cells were infected with a lentivirus-encoded shRNA to HIRA

(shHIRA) or control (shLuc) and stable cells were generated by drug selection with puromycin. Cells were treated with 2000U/ml of IFN-β and fluorescent stained with antibodies to PML and H3. **(H)** Quantitation of cells with H3 localized to PML bodies from (E). Data are mean +/- SEM from three independent experiments, $p<0.05$ comparing increase in H3 foci in shLuc vs increase in H3 foci in shHIRA after IFN-β. **(I)** Principal component analysis (PCA) of shLuc and shHIRA RNA-seq samples at 0h (red), 6h (green) and 24h (blue) after IFN-β treatment. The plot was generated based on expression of each known coding gene by FPKM. **(J)** Bar chart showing the proportion of genes that change in expression (FDR <= 5%) in shLuc between 0h-6h IFN-β or in shLuc between 6h-24h IFN-β, that have a concordant expression profile (i.e. same direction of expression change) upon HIRA knock down.

Figure S4

Control (*CAGG-Cre-ER*, WT, +tamoxifen) or Hira-deficient (*CAGG-Cre-ER*, *Hirafl/fl*, +tamoxifen) mice were infected with MCMV and liver harvested four days later. Plaque forming units (PFU) of MCMV recovered from liver samples of WT or *Hira*$^{-/-}$ mice were calculated as PFU/gram of tissue. Significance was assessed using the Mann Whitney-U test (n=9 for WT mice and n=18 for *Hira*$^{-/-}$mice).

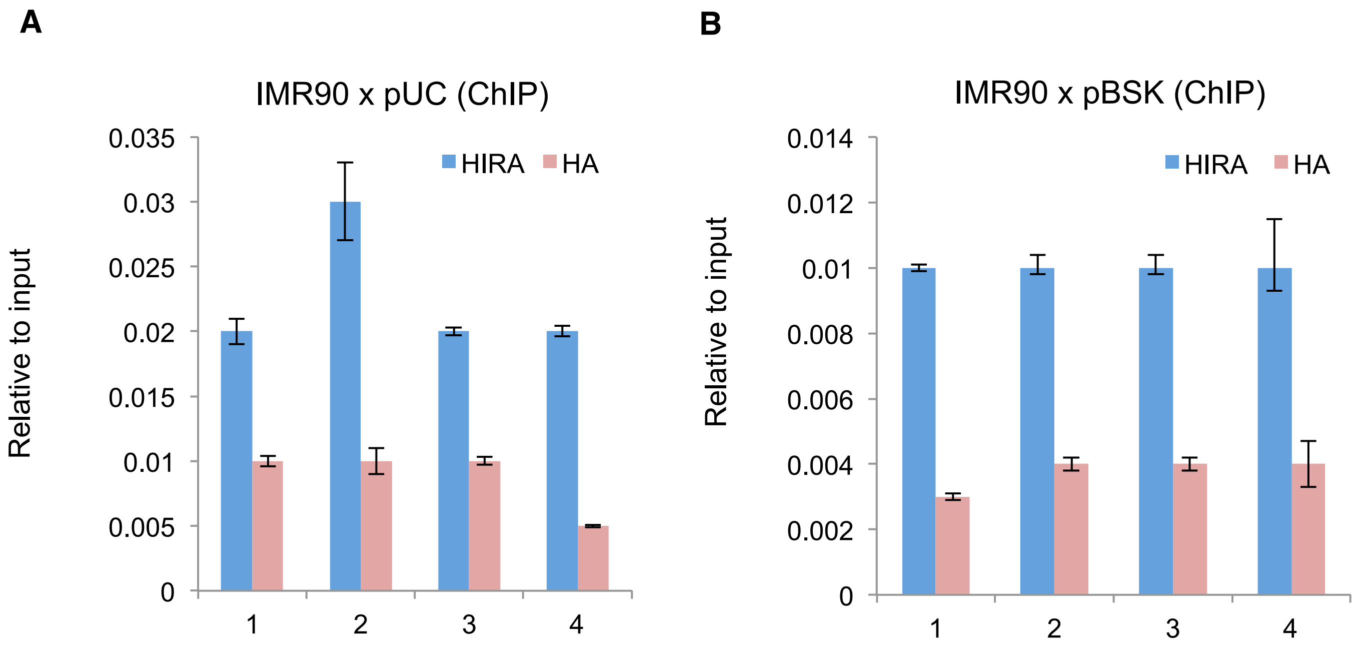
A
IMR90 x pUC (ChIP)
Relative to input
0.035
0.03
0.025
0.02
0.015
0.01
0.005
0
HIRA
HA
1
2
3
4
B
IMR90 x pBSK (ChIP)
Relative to input
0.014
0.012
0.01
0.008
0.006
0.004
0.002
0
HIRA
HA
1
2
3
4

Rai et al._Figure S2, related to Figure 2

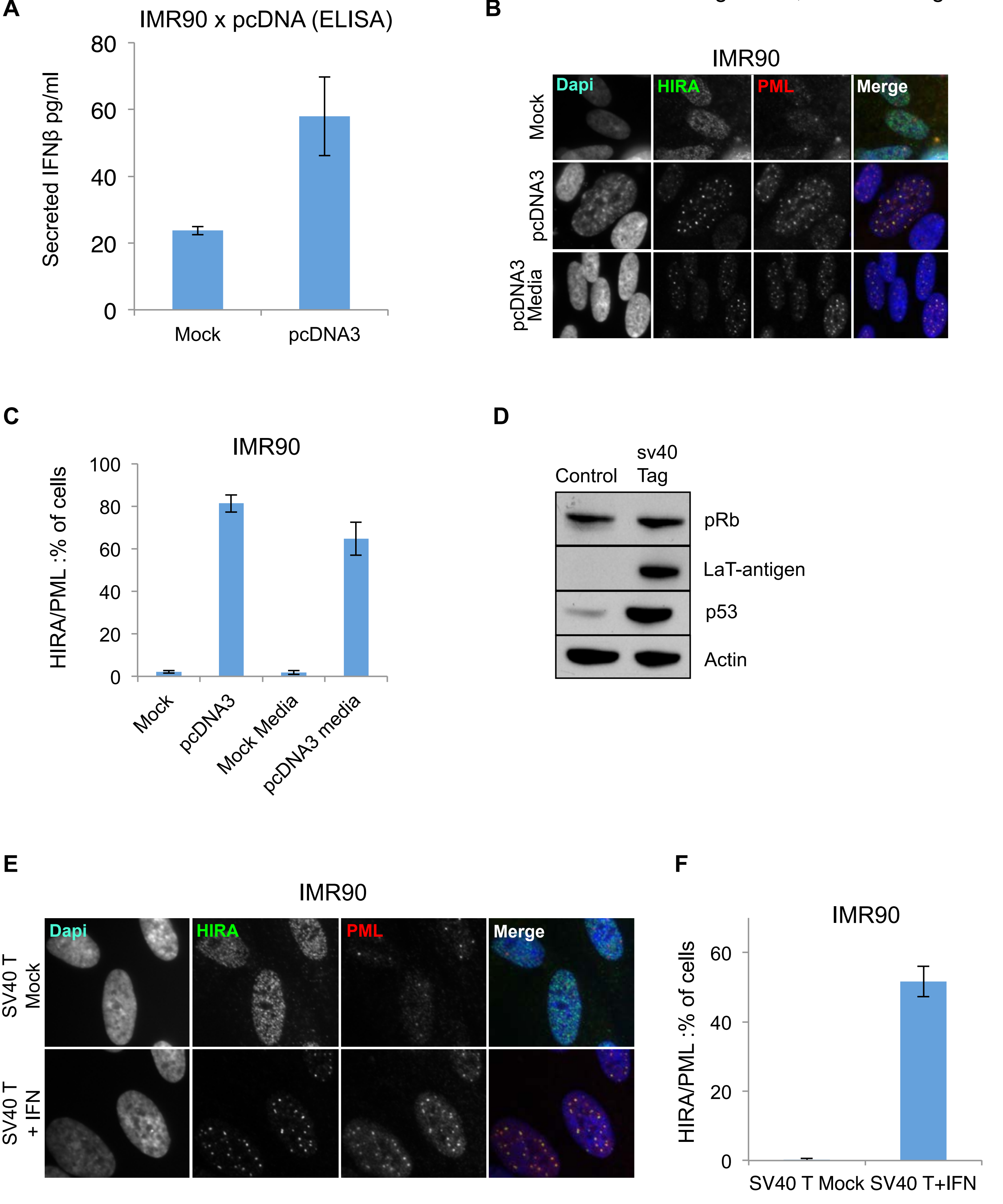

Rai et al._Figure S3, related to Figure 3

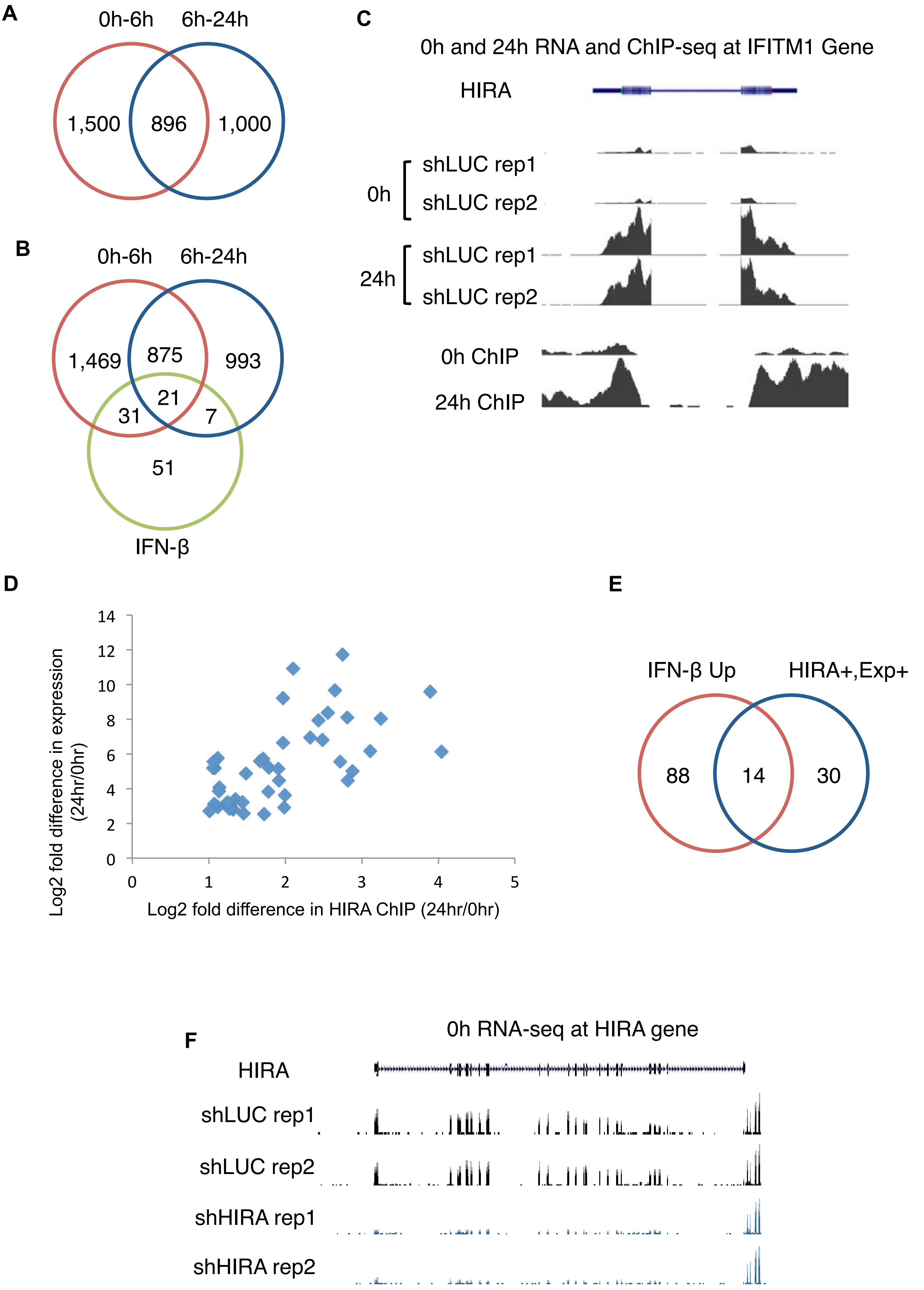

G

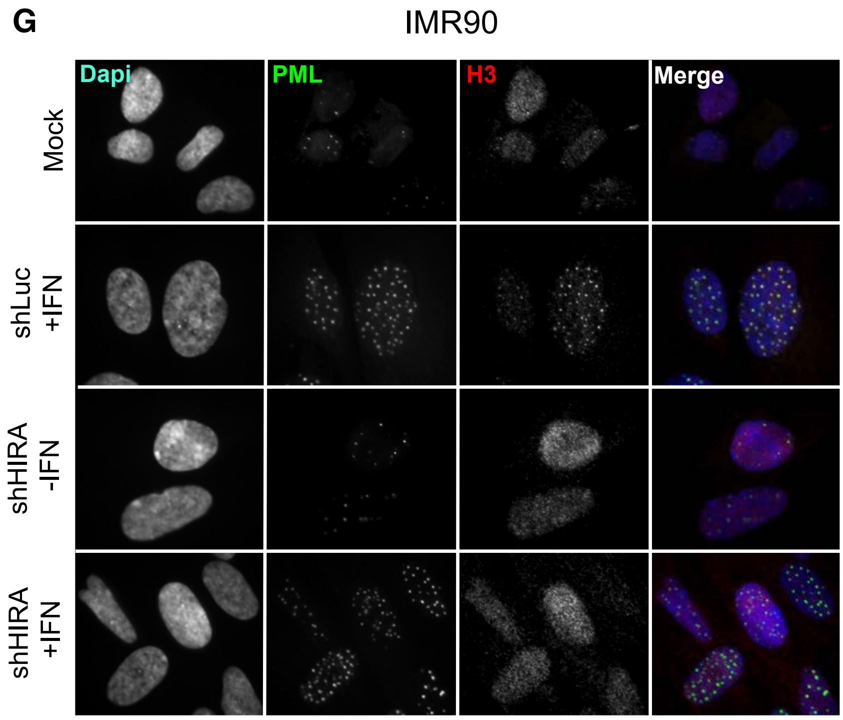
IMR90
Dapi
PML
H3
Merge
Mock
shLuc +IFN
shHIRA -IFN
shHIRA +IFN

H

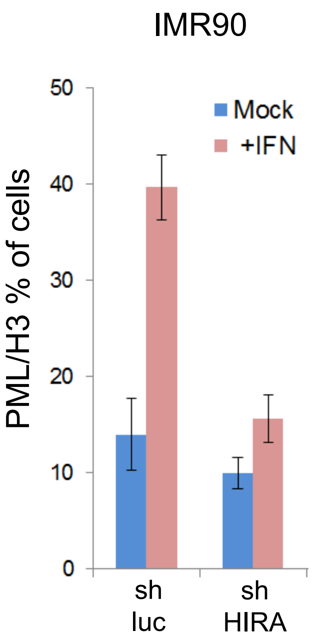
IMR90
PML/H3 % of cells
Mock
+IFN
50
40
30
20
10
0
sh luc
sh HIRA

I

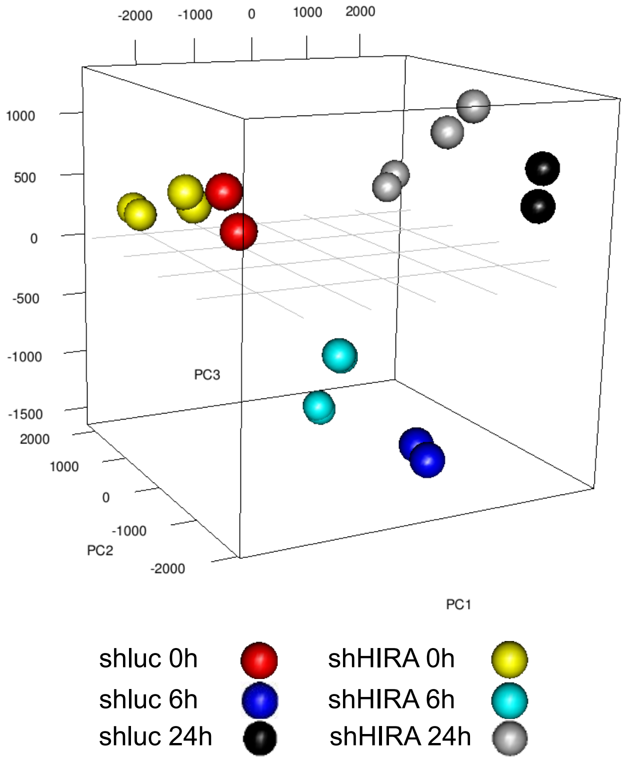
PC1
PC2
PC3
shluc 0h
shluc 6h
shluc 24h
shHIRA 0h
shHIRA 6h
shHIRA 24h

J

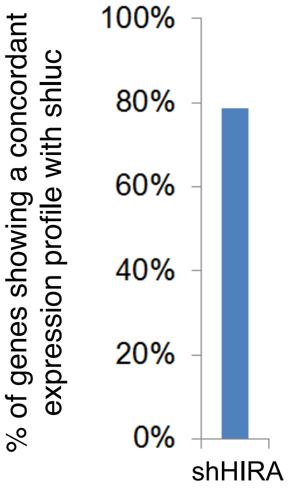
% of genes showing a concordant expression profile with shluc
100%
80%
60%
40%
20%
0%
shHIRA

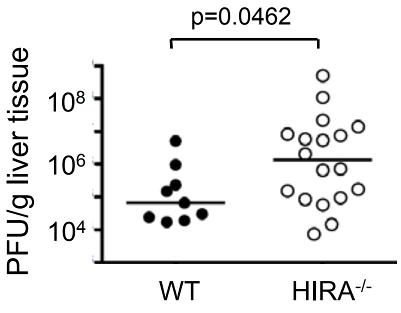
p=0.0462
PFU/g liver tissue
10^8
10^6
10^4
WT
HIRA-/-

1. Supplemental Tables and Data

Table S1. RNA sequencing

Sample	Timepoint	Replicate	Raw reads	Aligned reads (% of raw reads)	Non-duplicate reads (% of aligned reads)
shLUC	0	1	37,243,231	35,362,419 (94.95%)	30,803,490 (87.11%)
shLUC	0	2	40,109,120	38,330,377 (95.57%)	33,057,866 (86.24%)
shLUC	6	1	39,568,364	37,921,837 (95.84%)	32,549,338 (85.83%)
shLUC	6	2	41,879,610	40,271,287 (96.16%)	34,454,279 (85.56%)
shLUC	24	1	75,034,469	71,852,202 (95.76%)	61,435,186 (85.50%)
shLUC	24	2	85,794,160	81,961,651 (95.53%)	70,046,554 (85.46%)
shHIRA1	0	1	40,970,155	39,232,567 (95.76%)	32,264,776 (82.24%)
shHIRA1	0	2	38,309,633	36,583,400 (95.49%)	29,777,717 (81.40%)
shHIRA1	6	1	41,063,476	39,295,686 (95.69%)	32,116,387 (81.73%)
shHIRA1	6	2	40,300,138	38,550,705 (95.66%)	31,409,101 (81.47%)
shHIRA1	24	1	39,603,935	37,738,917 (95.29%)	30,637,502 (81.18%)
shHIRA1	24	2	78,388,480	74,748,607 (95.36%)	60,495,638 (80.93%)

Table S2. ChIP sequencing

Sample	Raw reads	Aligned reads (% of raw reads)	Uniquely Aligned Reads (% of Aligned Reads)	Duplicate Reads (% of Uniquely Aligned Reads)
HIRA IFN-	58,875,273	56,415,129 (95.82%)	41,774,709 (74.05%)	2,800,932 (6.70%)
HIRA IFN+	69,045,926	65,592,946 (95.00%)	47,064,100 (71.75%)	3,053,906 (6.49%)
HIRA IFN- Input	72,935,989	71,742,243 (98.36%)	54,554,023 (76.04%)	2,006,335 (3.68%)
HIRA IFN+ Input	32,549,655	32,038,804 (98.43%)	24,308,580 (75.87%)	854,717 (3.52%)

Table S3. primer sequences

pCDNA3F1	AACGCCAATAGGGACTTTCC
pCDNA3R1	GGGCGTACTTGGCATATGAT
pCDNA3F2	TAGTTGCCAGCCATCTGTTG
pCDNA3R2	GCGATGCAATTTCCTCATTT
pCDNA3F3	GGCGGTAATACGGTTATCCA
pCDNA3R3	TTTTTGTGATGCTCGTCAGG
pCDNA3F4	TTGCCGGGAAGCTAGAGTAA
pCDNA3R4	AAGCCATACCAAACGACGAG
pUC F1	GGTGTGAAATACCGCACAGA
pUC R1	CTGGCGTAATAGCGAAGAGG
pUC F2	GGCGCTTTCTCATAGCTCAC
pUC R2	GTCTTACCGGGTTGGACTCA
pUC F3	TTTGTTTGCAAGCAGCAGAT
pUC R3	CGTGAGTTTTCGTTCCACTG
pUC F4	TTGCCGGGAAGCTAGAGTAA
pUC R4	AAGCCATACCAAACGACGAG
pBSK F1	AATTTCCATTCGCCATTCAG
pBSK R1	AACGTCGTGACTGGGAAAAC
pBSK F2	GGCGGTAATACGGTTATCCA
pBSK R2	TTTTTGTGATGCTCGTCAGG

pBSK F3	GGCGCTTTCTCATAGCTCAC
pBSK R3	GTCTTACCGGGTTGGACTCA
pBSK F4	AAGTTGGCCGCAGTGTTATC
pBSK R4	CGCCGCATACACTATTCTCA
HSV F2	ACTTAATCAGGTTGTTGCCG
HSV R2	GAAGTTGTGGACTGGGAAGG
HSV F3	CTGGACGATAAGTCGGTGGAA
HSV F3	AGTGTGCTCGTCGTCTCCTGT
HSV F4	ATTAGCGTAAGGATGATGGT
HSV R4	AGACCTCAACGTGCTGTACT
HSV F5	CGCCGGTGTGTGATGATTT
HSV R5	TTTATACCCGGGCCCCAT
HSV F6	AGATCTGCGGCACGCTGTTG
HSV R6	CAGCTGCTTCATCCCCGTGG
